# Base and Prime Editing Technologies for Blood Disorders

**DOI:** 10.3389/fgeed.2021.618406

**Published:** 2021-01-28

**Authors:** Panagiotis Antoniou, Annarita Miccio, Mégane Brusson

**Affiliations:** Université de Paris, Imagine Institute, Laboratory of Chromatin and Gene Regulation During Development, INSERM UMR 1163, Paris, France

**Keywords:** genome editing, base editing, CRISPR/Cas9, genetic disorders, blood diseases

## Abstract

Nuclease-based genome editing strategies hold great promise for the treatment of blood disorders. However, a major drawback of these approaches is the generation of potentially harmful double strand breaks (DSBs). Base editing is a CRISPR-Cas9-based genome editing technology that allows the introduction of point mutations in the DNA without generating DSBs. Two major classes of base editors have been developed: cytidine base editors or CBEs allowing C>T conversions and adenine base editors or ABEs allowing A>G conversions. The scope of base editing tools has been extensively broadened, allowing higher efficiency, specificity, accessibility to previously inaccessible genetic loci and multiplexing, while maintaining a low rate of Insertions and Deletions (InDels). Base editing is a promising therapeutic strategy for genetic diseases caused by point mutations, such as many blood disorders and might be more effective than approaches based on homology-directed repair, which is moderately efficient in hematopoietic stem cells, the target cell population of many gene therapy approaches. In this review, we describe the development and evolution of the base editing system and its potential to correct blood disorders. We also discuss challenges of base editing approaches–including the delivery of base editors and the off-target events–and the advantages and disadvantages of base editing compared to classical genome editing strategies. Finally, we summarize the recent technologies that have further expanded the potential to correct genetic mutations, such as the novel base editing system allowing base transversions and the more versatile prime editing strategy.

## Introduction

The vast majority of human genetic diseases are due to point mutations. In fact, amongst the 54,444 human disease-causing variants described in ClinVar, 33,739 are point mutations (Rees and Liu, 2018).

Human blood genetic disorders are due to mutations affecting hematopoietic stem cells (HSCs) or their committed progeny leading to general hematopoiesis defects or lineage-specific damages (e.g., in leukocytes or erythrocytes). For example, β-hemoglobinopathies are due to >300 mutations affecting the β-globin gene (*HBB*), resulting in red blood cell (RBC) defects and anemia (Cavazzana et al., 2017; Amaya-Uribe et al., 2019). Allogeneic HSC transplantation is the only curative treatment for many blood genetic disorders. However, it is limited by the availability of sibling donors and is associated with risks of graft rejection and graft vs. host disease (Cavazzana et al., 2017; Castagnoli et al., 2019). Therefore, *ex vivo* gene therapy approaches based on autologous transplantation of genetically corrected HSCs have been developed to offer a permanent and safer therapeutic solution. Many clinical studies using lentiviral-based gene addition approaches have proven to be beneficial for patients with genetic blood disorders. Nevertheless, some limitations still exist; for example, the expression of the transgene might be insufficient to cure the disease. The CRISPR/Cas9 nuclease allows the correction of genetic mutations, therefore achieving a physiological expression of the target endogenous gene; however, it introduces double-strand breaks (DSBs) that can be deleterious for the target cells (Cromer et al., 2018; Kosicki et al., 2018).

Hematological malignancies have been successfully treated using chimeric antigen receptor (CAR) T-cell therapies. This approach is based on the engineering of autologous or allogenic T-cells that express a CAR recognizing antigens on tumor cells (e.g., CD19 in B-cell malignancies). In allogenic CAR T-cell therapies, several genes involved in alloreactivity can be inactivated using nuclease-based approaches. Nonetheless, DSBs can lead to genomic translocations, when simultaneous edits of different loci occur (Stadtmauer et al., 2020).

Base editing is a newly developed tool able to precisely edit DNA sequences in a specific locus without inducing DSBs. Interestingly, around 60% of the pathogenic point mutations can be potentially corrected by base editors (BEs) (Rees and Liu, 2018). Notably, base editing is a new therapeutic tool able to precisely and safely correct genetic mutations and to target disease modifiers and inactivate genes or *cis*-regulatory regions in hematopoietic cells. Therefore, base editing can potentially provide a cure for many blood diseases.

**Figure 1:**
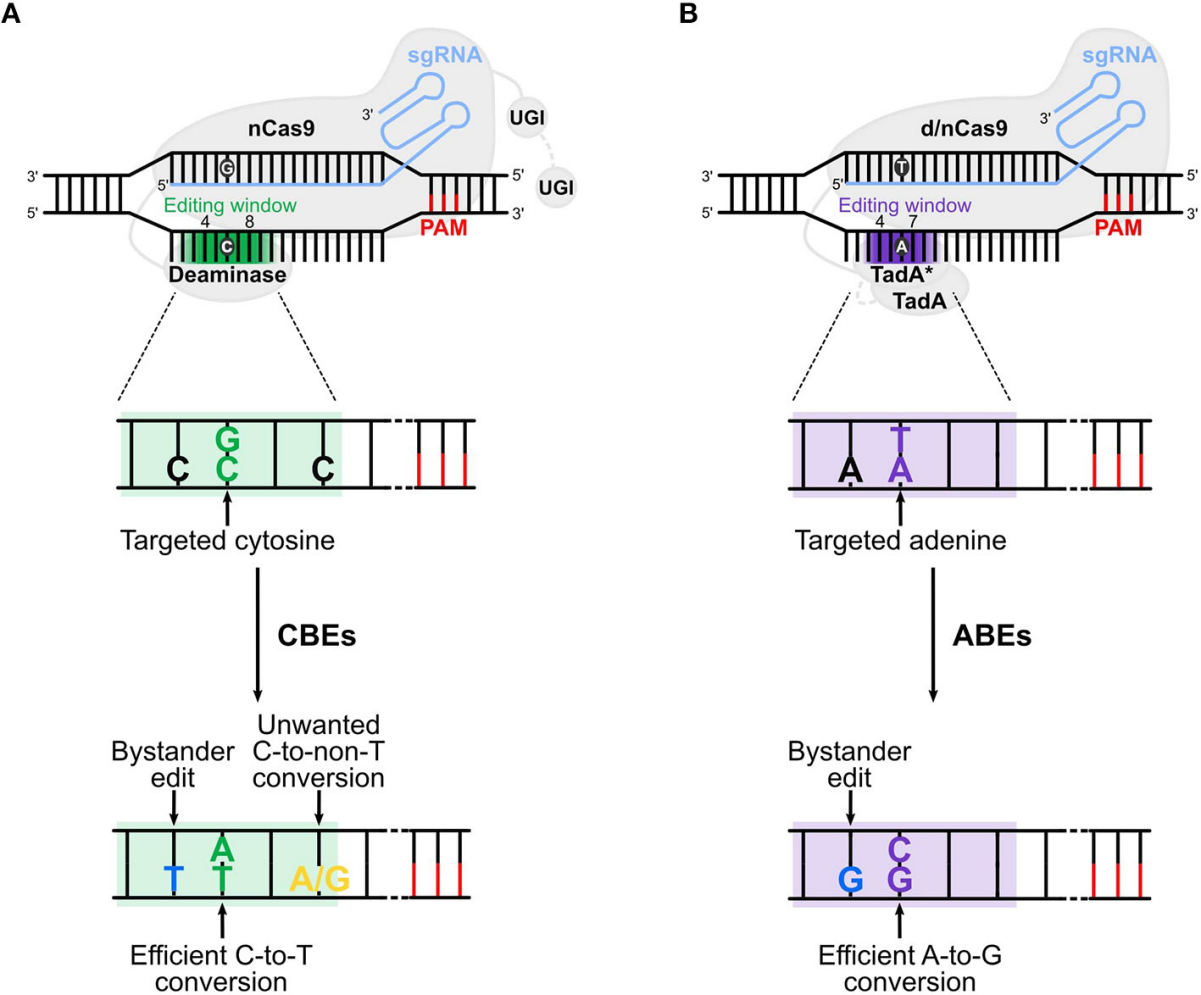
Cytosine and adenine base editors. **(A)** Cytosine base editors (CBEs), composed of a nickase Cas9 (nCas9) fused to a deaminase and one (in BE3s) or two (in BE4s) UGI (uracil glycosylase inhibitor), convert C:G into T:A base pairs in the editing window (nucleotide 4 to 8 in the protospacer, in green). **(B)** Adenine base editors (ABEs) are composed of a dead (d) or nickase (n) Cas9 (d/nCas9) fused to two TadA, one evolved to edit adenine in DNA (TadA*) and one wild type (TadA). ABEs convert A:T into G:C base pairs in the editing window (nucleotide 4 to 7 in the protospacer, in purple). Cas9 is guided by the sgRNA to the protospacer [which is followed by the PAM (protospacer adjacent motif)], unwinds the DNA and the deaminase converts the target base. Undesired events (bystander edits, in blue, and unwanted base conversion, in yellow) of CBEs and ABEs are shown in **(A,B)**, respectively. The addition of the second UGI in CBEs (in BE4) and the removal of TadA in ABEs (ABE8) are highlighted with a gray dotted line. The gradient color of the editing window in the upper panels of **(A,B)** represents the enlarged editing window observed with novel BEs.

Different BEs have been created allowing base conversions in a variety of target regions. The cytosine BEs (CBEs) allow the conversion of a C:G to a T:A base pair (bp), while adenine BEs (ABEs) convert an A:T into a G:C bp. BEs are composed by a catalytically dead Cas9 (dCas9) or a nickase Cas9 (nCas9) fused to a deaminase and guided by a single guide RNA (sgRNA) to the locus of interest (Figure 1). The d/nCas9 recognizes a specific sequence named protospacer adjacent motif (PAM) and the DNA unwinds thanks to the complementarity between the sgRNA and the DNA sequence usually located upstream of the PAM (“protospacer”). Then, the opposite DNA strand is accessible to the deaminase that converts the bases located in a specific DNA stretch of the protospacer (“editing window,” Figure 1).

One of the major advantages of BEs compared to the CRISPR/Cas9 nuclease system is their ability to introduce precise point mutations without generating DSBs. In fact, despite the high efficiency, CRISPR/Cas9 treatment of human hematopoietic stem/progenitor cells (HSPCs) induces a DNA damage response (Cromer et al., 2018) that can lead to apoptosis. CRISPR/Cas9 can cause P53-dependent cell toxicity (Haapaniemi et al., 2018; Ihry et al., 2018; Schiroli et al., 2019) and cell cycle arrest, resulting in negative selection of cells with a functional P53 pathway. Furthermore, the generation of several on-target DSBs, simultaneous on-target and off-target DSBs, or even a single on-target DSB is associated with a risk of deletion, inversion, and translocation (Kosicki et al., 2018; Cullot et al., 2019; Blattner et al., 2020; Leibowitz et al., 2020). These events impair gene correction and might result in the complete inactivation of the target gene or even have long-range transcriptional consequences that could constitute a first carcinogenic hit. Therefore, the absence or the very low frequency of DSBs, confer to BEs the potential to perform safer genome edits. Moreover, BEs accurately convert specific bases in a wide range of cell types and at different stages along the cell cycle. On the contrary, nuclease-based correction of genetic mutations via homology-directed repair (HDR) is limited mainly to dividing cells (Zhang et al., 2017). Compared to HDR-based strategies, base editing is a promising therapeutic tool to precisely correct genetic mutations as it avoids gene disruption by non-homologous end-joining (NHEJ) associated with failed HDR-mediated gene correction (Yeh et al., 2018). Finally, this DSB-free strategy can potentially allow simultaneous targeting of multiple regions in the genome without generating chromosomal rearrangements such as large deletions and translocations (Stadtmauer et al., 2020).

## Development of Cytosine and Adenine Base Editors

Different versions of CBEs have been created with the goal of improving their efficiency and safety. The original BE1 is composed of a catalytically dCas9 from *Streptococcus pyogenes (Sp)* fused with the rat deaminase (rAPOBEC1). This enzyme was selected amongst several deaminases for its high deaminase activity (Komor et al., 2016). The dCas9 contains amino acid substitutions (D10A and H840A) that abolish the nuclease activity avoiding DSB generation without interfering with its DNA binding capacity. BE1 recognizes the cytosine at the target locus and converts it into a uracil. The U:G bp is recognized as a mismatch by the cellular repair machinery that usually removes the U. To protect this newly formed U from excision, BE2 was developed by fusing a uracil glycosylase inhibitor (UGI) to the dCas9 C-terminus (Komor et al., 2016). In BE3, the dCas9 was modified to generate a Cas9 nickase (Cas9n containing the D10A amino acid substitution) that nicks the non-edited G-containing DNA strand without generating DSBs (Komor et al., 2016). The nicking step favors the replacement of the G in the nicked strand by an A by the DNA repair machinery. Then, the uracil from the U:A bp is converted to T by the host repair machinery allowing the formation of the desired T:A bp. These modifications improved the efficiency of CBEs in mammalian cells (Komor et al., 2016). Finally, the fourth-generation BE4 differs from BE3 as it carries a second UGI conferring a higher editing efficiency and improved product purity (percentage of C converted to T over the total base conversion events (C>T, C>G, and C>A) (Komor et al., 2017). The editing window of these CBEs is located at positions 4–8 of the protospacer (with the PAM's first nucleotide located at position 21). The use of alternative cytosine deaminases was also explored, such as the *P.marinus* activation-induced cytidine deaminase (AID or PmCDA1; editing window at positions 2–8) and the human APOBEC3A (hA3A; editing window at positions 2–13) (Gehrke et al., 2018; Nishimasu et al., 2018; Wang et al., 2018).

The absence of a DNA adenine deaminase to target and convert an A:T bp to a G:C bp prompted Liu and coworkers to create an engineered enzyme (Gaudelli et al., 2017). A dimeric tRNA adenine deaminase from *E. coli* (TadA) was modified to generate TadA^*^ that efficiently deaminates adenine in the DNA. TadA* was then fused to the SpCas9n (D10A) to create ABEs. As TadA works natively as a homodimer, an enzyme composed of wild-type TadA and TadA^*^ was fused to SpCas9n and various mutations were introduced in the TadA^*^ domain. This resulted in the development of four ABEs (ABE6.3, ABE7.8, ABE7.9, and ABE7.10) with increased editing efficiency. The editing window is located at positions 8-10 of the protospacer for ABE6.3, ABE7.8, and ABE7.9 and at position 4–7 for ABE7.10. Therefore, for the same sgRNA, the choice of the ABE can be dictated by the position of the target bases (Gaudelli et al., 2017). Interestingly, at the same loci, ABEs were able to introduce point mutations with a higher efficiency and reduced InDel formation compared to Cas9 nuclease-mediated HDR approaches (Gaudelli et al., 2017).

BE3, BE4, and ABE7.10 are the most commonly used base editors nowadays and have been extensively improved in the last years (Gaudelli et al., 2020; Miller et al., 2020; Richter et al., 2020). These base editors have been optimized by modifying the codon usage and the nuclear localization sequences to enhance base editing in mammalian cells [e.g., BE4max, AncBE4max, and ABEmax (Koblan et al., 2018)]. For instance, BE4 was improved by the addition of a bipartite NLS at both N- and C-termini and by codon optimization to generate BE4max. Replacement of rAPOBEC1 with an optimized ancestor rAPOBEC1 homolog—Anc689 that contains 36 amino acid substitutions compared to rAPOBEC1–resulted in the generation of AncBE4max. Both BE4max and AncBE4max exhibit a higher editing efficiency compared to BE4 (Koblan et al., 2018). Furthermore, the use of alternative Cas variants or the engineering of Cas enzymes allowed the development of BEs recognizing a greater variety of PAMs, thus expanding the targeting scope of BEs. Finally, modifications of the deaminase domain led to the generation of more precise BEs with increased product purity and a narrower activity window.

### Improving the Targeting Scope of Base Editors

One of the limitations to the use of BEs is the requirement of a suitable PAM adjacent to the target sequence and in a position that places the target bases in the optimal editing window. The first CBEs and ABEs were designed using the SpCas9n (that is the most commonly used Cas for genome editing) limiting the editing to genomic loci containing NGG PAMs. To increase the number of potential targets, BEs harboring orthologous Cas9n or engineered Cas9n variants have been developed. These enzymes recognize non-NGG PAMs and for some of them, the editing window is shifted or enlarged to target bases that otherwise would be inaccessible due to the lack of an optimal PAM. Finally, the use of alternative or engineered deaminase variants was also explored to enlarge the editing window.

#### CBEs With Expanded Targeting Range

To broaden the targeting scope of CBEs, new Cas9n variants have been introduced in CBEs allowing the editing of non-NGG PAM sites.

BE3s harboring the engineered SpCas9n variants SpCas9n-VQR (NGA PAM), SpCas9n-VRQR (NGA PAM), SpCas9n-EQR (NGAG PAM), and SpCas9n-VRER (NGCG PAM) allowed the targeting of genomic regions containing non-NGG PAMs (Kim et al., 2017b).

Furthermore, Kim et al. created SaBE3 harboring the nickase version of the *Staphylococcus aureus* Cas9 (SaCas9n, containing the D10A amino acid substitution), which edits sites containing NNGRRT PAMs (Ran et al., 2015). SaBE3 effectively converts C to T in human cells with a high conversion efficiency at NNGRRT PAM compared to BE3 (Kim et al., 2017b). The SaCas9n was also introduced in BE4 to create SaBE4, resulting in higher editing efficiency and product purity compared to SaBE3 (Komor et al., 2017). A SaCas9n mutant harboring three mutations (SaCas9n-KKH, SaKKHn) was used to develop CBEs that can target loci containing NNNRRT PAMs (Kim et al., 2017b). Importantly, Sa BEs have an expanded editing window compared to Sp BEs (positions 3–12) allowing the editing of bases located closer to the PAM.

Interestingly, a small Cas9 nickase from *Staphylococcus auricularis* (SauriCas9n containing the D15A amino acid substitution) was inserted in the BE4max (SauriBE4max). Its reduced size allowed the packaging in adeno-associated virus (AAV) vectors. In addition, this novel CBE allows the targeting of loci containing NNGG PAMs (Hu et al., 2020).

To enlarge the number of editable loci that lack G/C-rich PAM sequences, Li et al. fused the dead Cas12a from *Lachnospiraceae bacterium* (dLbCas12a or dLbCpf1) to rAPOBEC1 to generate dCas12a-BE3. This CBE can edit loci containing T-rich PAMs (TTTV) and efficiently convert cytosines located downstream of the PAM (from position 8–13, counting the base next to the PAM as position 1) with minimal InDels and undesired base conversions (Li et al., 2018b). Another engineered Cas12a variant from *Acidaminococcus sp*. (enAsCas12a) was used to generate CBEs that recognize TTTV as well as additional PAMs (e.g., TTYN, VTTV TRTV). These BEs show improved C>T conversion compared to the original AsCas12a (Kleinstiver et al., 2019). Finally, the insertion of an engineered SpCas9n containing the PAM-interacting region of *Streptococcus macacae* Cas9 (Spy-macCas9n) in BE4max led to the development of Spy-mac-BE4max, which is capable of targeting sites containing TAAA PAMs (Liu et al., 2019).

To further increase the number of genomic regions accessible to CBEs, Hu et al. developed new SpCas9 variants harboring mutations that expand the PAM compatibility. In particular, the use of the xCas9 variant recognizing a large range of PAMs (including NG, GAA, and GAT) in the BE3 enzyme (xCas9-BE3) greatly increased cytosine base editing scope. However, this BE was proved efficient in a limited number of genomic sites (Hu et al., 2018). Another engineered Cas9 variant recognizing NG PAMs [SpCas9n-NG (Nishimasu et al., 2018)] was incorporated in CBEs harboring rAPOBEC1, APOBEC3A or PmCDA1 (Nishimasu et al., 2018; Thuronyi et al., 2019). In particular, Nishimasu et al. showed that a fusion of PmAID and SpCas9n-NG (Target-AID-NG) is more active than the xCas9-BEs in human cells (Nishimasu et al., 2018). Recently, novel CBEs compatible with NRCH, NRTH or NRRH PAMs (including non-G PAMs) allowed the targeting of previously inaccessible genomic loci (Miller et al., 2020).

Besides expanding the PAM compatibility of CBEs, several studies aimed at targeting cytosines outside the classical editing window. A larger editing window can allow the installation of point mutations in previously inaccessible regions to disrupt genes or regulatory regions; however, if the goal is to generate a precise mutation (e.g., in the coding region of a gene), only silent mutations of non-target bases should be permitted. Huang et al. generated novel circularly permutated (CP)-SpCas9n BE variants with a broadened or shifted editing window. These variants were used to generate CP-BE4max enzymes that can efficiently edit bases that otherwise would be inaccessible (Huang et al., 2019). Interestingly, the introduction of the RAD51 single-stranded DNA binding domain (ssDBD) in BE4max, dramatically increased the editing frequency and extended the editing window to cytosines in positions 9–15 (hyBE4max) (Zhang et al., 2020a). Similar results were obtained by inserting the RAD51 ssDBD in BE4max harboring APOBEC3A (hyA3A-BE4max) (Zhang et al., 2020a).

Different deaminase variants can also be employed to target previously inaccessible sites. The introduction of human APOBEC3A (hA3A) deaminase in BE3 (hA3A-BE3) improved C-to-T base conversion in highly methylated genomic regions and enlarged the editing window to 12 nucleotides (position 2–13 in the protospacer) (Wang et al., 2018). Thuronyi et al. generated several rAPOBEC1 and PmCDA1 variants with improved context compatibility (i.e., allowing editing of GC motifs) and enlarged editing window. EvoFERNY (an ancestor of rAPOBEC1), evoAPOBEC1 (a rAPOBEC1 variant) and evoCDA1 (a PmCDA1 deaminase variant) deaminases were introduced in BE4max to test their activity in GC motifs, which were usually poorly edited by the previously developed CBEs. EvoCDA1-BE4max and evoFERNY-BE4max outperformed evoAPOBEC1-BE4max at GC target sites, while offering similar or even higher efficiency in non-GC targets. An advantage of evoFERNY-BE4max is the smaller size of its deaminase allowing its delivery by viral particles. While evoFERNY-BE4max and evoAPOBEC1-BE4max present an editing window comparable to BE4max, evoCDA1-BE4max offers an enlarged editing window (position 1–13 of the protospacer) and enables the conversion of cytosines located in GC or TC motifs far from the classical editing window (Thuronyi et al., 2019).

#### ABEs With Expanded Targeting Range

The use of orthologous or engineered Cas9n in ABEs increased the number of PAMs compatible with these enzymes, thus broadening the range of adenine base editing targets.

Several Cas9n variants were introduced in ABEs to generate A>G conversions at genomic sites containing non-NGG PAMs (Chatterjee et al., 2018; Hu et al., 2018; Yang et al., 2018; Jeong et al., 2019). SpCas9n was replaced by SaKKHn or SpCas9n-VQR in ABE7.10 to generate SaKKH-ABE and VQR-ABE that target sites harboring the NNNRRT and the NGA PAM, respectively (Yang et al., 2018). Similarly, Hu et al. introduced xCas9 in ABE7.10 and created xCas9-ABE offering improved editing efficiency at NGG PAM-containing sites as well as at loci harboring NGC, NGA, and GAT PAMs (Hu et al., 2018). ABEmax versions containing Cas9 variants recognizing NG (xCas9 in xABEmax or SpCas9n-NG in NG-ABE max) or NR PAMs (SpCas9n-NRCH, SpCas9n-NRTH, and SpCas9n-NRRH) have also been generated (Huang et al., 2019; Miller et al., 2020). ABEmax was further improved by replacing SpCas9n with SaCas9n or with the engineered SaKKHn, SpCas9n-VRER and SpCas9n-VRQR allowing the targeting of loci containing non-NGG PAMs. SpCas9n-VRER and SpCas9n-VRQR induce A-to-G conversions in many target sites containing PAMs other than NGG. Sa-ABEmax and SaKKH-ABEmax present a large editing window (position 4–14 of the protospacer) although the editing efficiency is modest (Huang et al., 2019). To target bases located outside the canonical editing window, Huang et al. generated CP-ABEmax enzymes with a broadened or shifted activity window (Huang et al., 2019).

Recently, Richter et al. developed a novel ABE (ABE8e) with enhanced activity and compatibility with different Cas homologs, which was limited with the previously described ABEs (Richter et al., 2020). ABE8e contains eight additional mutations in the TadA^*^ deaminase domain that confer a higher processing activity (Lapinaite et al., 2020). ABE8e showed greatly increased editing efficiency when combined with SpCas9n and different Cas9 variants (e.g., SaCas9n, SaKKHn, SpCas9n-NG, and LbCas12a) compared to the corresponding ABEmax-based enzymes (Richter et al., 2020). Furthermore, removal of the wild type TadA did not affect ABE8e editing activity, indicating that the optimized TadA^*^ can efficiently work as a monomer (Richter et al., 2020). Interestingly, Gaudelli et al. also generated ABE8 variants offering improved editing efficiency and extended editing window (position 3–10) compared to ABEmax (Gaudelli et al., 2020). The SpCas9n of ABE8s was replaced by the engineered SpCas9n-NG or SaCas9n, broadening the editing scope of ABE8s, while maintaining their preference for adenine editing in a wide editing window (position 5–14) (Gaudelli et al., 2020). Of note, ABE8 enzymes showed increased DNA and RNA off-target activity. However, this was reduced by delivering the BE as mRNA or ribonucleoprotein (RNP) (compared to plasmid delivery) or by inserting amino acid substitutions that enhance the genome-wide specificity (see paragraph “BE off-target activity”) (Gaudelli et al., 2020; Richter et al., 2020).

#### Virtually PAMless CBEs and ABEs

In a recent study, Walton et al. used structure-guided engineering to relax the PAM requirement of SpCas9, resulting in a near-PAMless variant (SpRY). SpRY is compatible with both CBEs and ABEs and with nearly all the possible PAMs (NRN and NYN, with low but substantial activity with NYN) (Walton et al., 2020). As expected, the PAM relaxation reduced specificity and increased the number of DNA off-targets (Walton et al., 2020). However, insertion of amino acid substitutions conferring a reduced or absent off-target activity can be envisioned to improve the precision of SpRY-based BEs (see paragraph “BE off-target activity”).

### Improving Product Purity of BEs

Base editing is a powerful tool to efficiently correct point mutations at a specific locus. However, at certain genomic targets, CBEs and, to a lesser extent, ABEs generate unwanted base conversions, thus reducing the product purity. In fact, the initial study describing CBEs reports that BE3 generates unwanted C>non-T edits inside the activity window (Komor et al., 2016). In a second study, Komor et al. improved BE3 by inserting a second UGI and by increasing the length of the linkers between rAPOBEC1 and Cas9n (32 amino acids), between Cas9n and UGI (9 amino acids), and between the 2 UGI (9 amino acids) (Komor et al., 2017). The new BE4 enzyme showed improved C-to-T editing efficiency and product purity and decreased InDel formation compared to BE3. Moreover, the introduction of CP-SpCas9 in BE4max to generate CP-BE4max improved product purity compared to BE4max (Huang et al., 2019). Finally, the Gam protein of the Mu bacteriophage, known to protect DSB ends from degradation, was fused to the N-terminal part of BE3, SaBE3, BE4, and SaBE4 via a linker of 16 amino acids. These four novel enzymes displayed lower InDel frequency and increased product purity without affecting C>T editing efficiency compared to their unmodified versions (Komor et al., 2017). Interestingly, the product purity was particularly high in human *bona fide* HSCs–the target cell population in gene therapy approaches for hematological genetic disorders (Zeng et al., 2020).

Contrary to CBEs, ABEs have a high product purity. Only one study describes ABEs as a generator of aberrant edits. Surprisingly, ABEs was not responsible for A>non-G edits but for C>G or C>T conversions (Kim et al., 2019).

### Reducing Bystander Edits and Narrowing the Activity Window of BEs

The vast majority of BEs convert cytosines or adenines located in a precise editing window of 4 to 6 nucleotides. The original BEs (BE3, BE4, and ABE7.10) display an editing window ranging from position 4–8 for CBEs and 4–7 for ABEs (Figure 1). However, if multiple C or A are present in the editing window, their conversion by BEs can potentially introduce undesired mutations (Komor et al., 2016). These bystander edits should be taken into consideration when base editing is used as a therapeutic strategy because they could create aberrant gene variants. Deaminase engineering was mainly used to narrow or shift the editing window and reduce bystander edits.

In the case of rAPOBEC1-based CBEs, mutations have been inserted in rAPOBEC1 to develop new BEs that precisely edit specific cytosines in the protospacer without modifying adjacent cytosines. Among all the mutants tested, the triple mutant YEE-BE3 (W90Y, R126E, and R132E) exhibits a restricted editing window of 1 to 2 nucleotides mainly editing cytosine at position 6 in the protospacer. If two C are present in the editing window, YEE-BE3 favors the conversion of only one of them (Kim et al., 2017b). However, this engineered BE displays reduced editing efficiency. Similarly, the introduction of the YEE mutations in the deaminase of BE4-Gam (YEE-BE4-Gam) and BE4max (YEE-BE4max) narrows the editing window to position 5 or 6 but lowers the editing efficiency (Liu et al., 2020). By removing the R132E mutation (known to reduce the editing efficiency) and introducing the Y120F mutation (known to narrow the editing window), Liu et al. created YFE-BE4max that presents a restricted editing window (position 4–6) and a high editing efficiency (Liu et al., 2020). The YE mutations were also introduced in the dCas12a-BE (dCas12a-BE-YE) to narrow the width of the editing window from 6 to 3 nucleotides (position 10-12 of the protospacer counting the base next to the PAM as position 1) (Li et al., 2018b).

Interestingly, the substitution of the original flexible linker between rAPOBEC1 and Cas9n by a rigid linker of 5–7 amino acids in BE3 greatly shortens the editing window and favors editing at positions 5 and 7 (Tan et al., 2019). Furthermore, truncation of PmCDA1 in the BE3-based enzyme restricted the editing window to position 2 (Tan et al., 2019).

Finally, to restrict the editing window and reduce the high InDel frequency associated with hA3A-BE3, Y130F, or Y132D mutations (known to partially reduce hA3A activity) and 3 UGI were inserted in hA3A-BE3 to generate BEs with a narrowed editing window (position 3–8) and lower InDel frequency (Wang et al., 2018). Gehrke et al. also introduced in BE3 an engineered hA3A (eA3A) containing the N57G amino acid substitution that improved the editing precision. This engineered eA3A-BE3 favors conversion of the C located in a TCR motif and reduces bystander mutations compared to BE3 and YE-BE3 variants (Gehrke et al., 2018).

Concerning the ABEs, no variant with a narrower editing window has been described up to date.

## Base Editing for the Treatment of Blood Disorders

### Base Editing Strategies for β-Hemoglobinopathies

β-hemoglobinopathies, β-thalassemia, and sickle cell disease (SCD), are monogenic diseases caused by mutations in the β-globin locus and affect the synthesis, the structure or the properties of the adult hemoglobin (HbA). β-thalassemia is caused by mutations in the β-globin locus that reduce (β^+^) or abolish (β^0^) the production of adult β-globin chains composing the HbA tetramer. This leads to the precipitation of uncoupled α-globin chains, ineffective erythropoiesis, erythroid cell death, and anemia (Weatherall, 2001; Cappellini et al., 2018; Taher et al., 2018). In SCD, an A>T mutation in the *HBB* gene causes the substitution of valine for glutamic acid at position 6 of the β-globin chain (β^S^) that is responsible for deoxygenation-induced polymerization of the sickle hemoglobin (HbS). This primary event drives RBC sickling, hemolysis, vaso-occlusive crises, multi-organ damage, often associated with severely reduced life expectancy (Piel et al., 2017; Kato et al., 2018).

**Figure 2:**
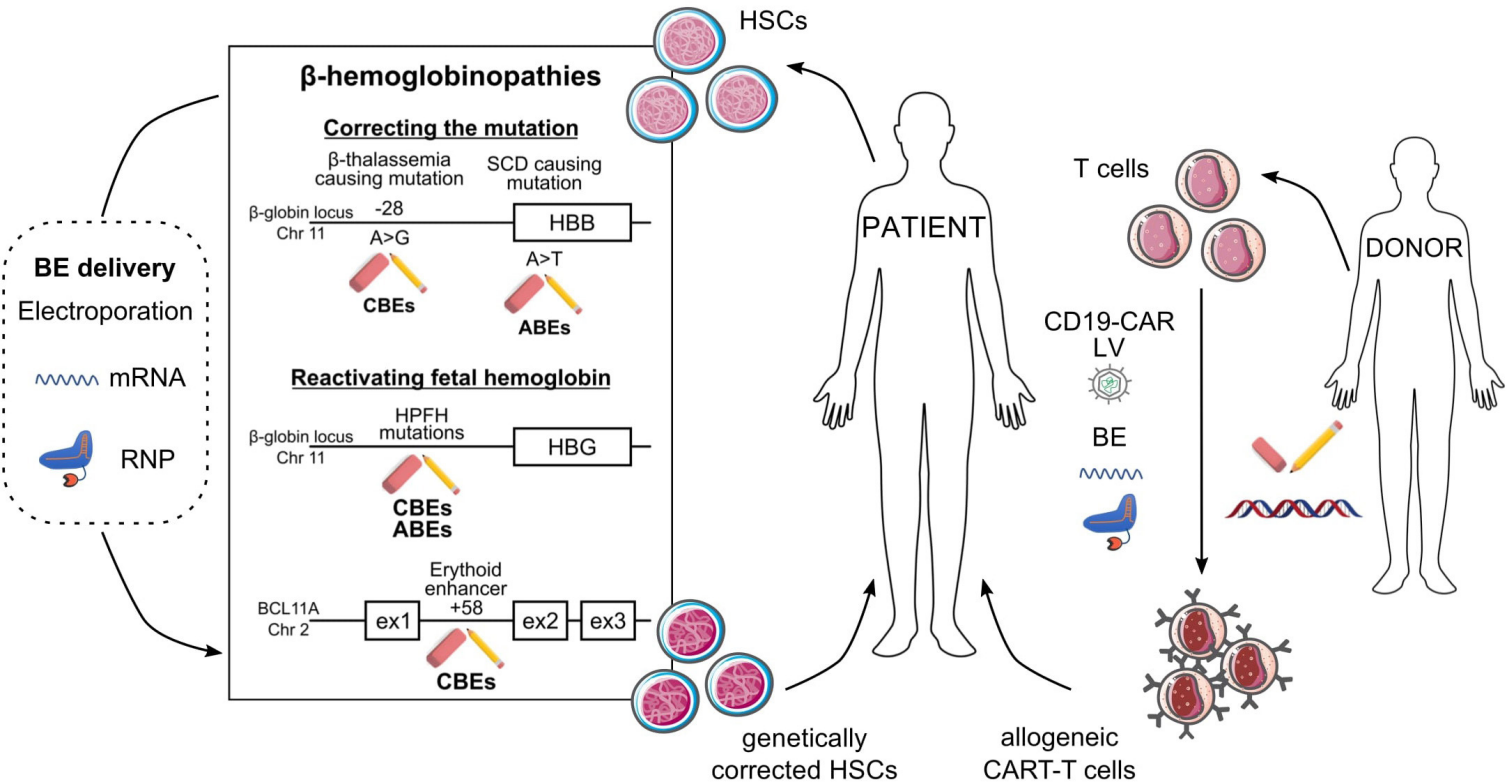
Potential *ex vivo* base editing approaches for genetic blood disorders. Schematic representation of base editing approaches to genetically correct HSCs from SCD and β-thalassemia patients (left) or to generate allogeneic CAR-T cells (right). (Left) Correction of the A>G β-thalassemic mutation (in position-28) and reversion of the SCD A>T mutation can be performed using CBEs and ABEs, respectively. HbF reactivation can be achieved (i) upon generation of HPFH mutations in *HBG1/2* promoters by ABEs or CBEs or (ii) upon disruption of the *BCL11A* erythroid enhancer (located at position +58 kb from *BCL11A* transcription start site) by CBEs. BEs are delivered to HSCs as mRNA or RNP complexes. In *ex vivo* gene therapy approaches, HSCs genetically modified by BEs will be transplanted to the patient as a definitive therapy. (Right) Multiplex base editing of loci involved in alloreactivity (e.g., *TRAC, B2M, PDC1D, CD7*) and lentiviral vectors (LV)-mediated CAR expression to safely generate allogeneic CAR-T cells, which will be infused into patients to kill cancer cells.

Allogenic HSC transplantation is the only curative therapy for β-hemoglobinopathies; however, the absence of sibling donors and the risk of immunological complications prevent its use in a large fraction of patients (Locatelli et al., 2013, 2016; Leonard and Tisdale, 2018). Because of their high prevalence, β-hemoglobinopathies are a common study model for developing genetic treatments. Transplantation of lentiviral-corrected HSCs containing a functional β-globin gene is a promising therapeutic solution for patients lacking sibling donors. However, the low expression level of the therapeutic transgene per viral copy is associated with a variable clinical outcome (Miccio et al., 2008; Thompson et al., 2018; Weber et al., 2018; Cavazzana et al., 2019; Magrin et al., 2019; Marktel et al., 2019). Promising genome editing-based therapies were developed to directly modify endogenous genes and induce therapeutic β-like globin expression. Current nuclease-based strategies can reactivate the expression of fetal γ-globin genes or correct the defective β-globin gene. CRISPR/Cas9-mediated editing strategies raising γ-globin levels take advantage of the NHEJ pathway to disrupt genes or *cis*-regulatory regions involved in γ-globin silencing. As NHEJ is an active DNA repair pathway in HSCs, NHEJ-based strategies are highly efficient (Wu et al., 2019; Weber et al., 2020). On the contrary, HDR-based approaches are modestly efficient in quiescent HSCs. For instance, the SCD-causing mutation was efficiently corrected by CRISPR/Cas9 combined with a donor template in HSPCs. However, the efficiency of gene correction was drastically reduced after xenotransplantation in immunodeficient mice, confirming the low HDR rate in long-term repopulating HSCs (Dever et al., 2016; Antony et al., 2018; Pattabhi et al., 2019; Romero et al., 2019). Finally, one of the major limitations of nuclease-based approaches is the potential DSB-induced toxicity (see introduction). Therefore, BEs could provide safer therapeutic strategies (Figure 2, Table 1). Notably, these approaches could be more efficacious than HDR-based strategies in correcting β-hemoglobinopathy-causing mutations, as base editing is efficient in quiescent cells, as are HSCs (Zeng et al., 2020).

**Table 1:** Base editing strategies for the treatment of blood disorders.

		**Model**	**Delivery**	**Target**	**BE**	**Efficiency**	**References**
β-hemoglobinopathies	Cell line	β^S/^β^S^ HEK293T cells	Plasmid chemical transfection	SCD mutation	ABE-NRCH	41%	Miller et al., 2020
		HEK293T cells		−198 *HBG1/2* (HPFH)	ABE7.10	30%	Gaudelli et al., 2017
		HEK293T cells		−175/−113/−116 *HBG1/2* (HPFH)	ABEmax	27–52%	Koblan et al., 2018
		HEK293T cells		−198 and −175 *HBG1/2* (HPFH)	ABE8e	24%	Richter et al., 2020
		HEK293T cells		Erythroid-specific *BCL11A* enhancer	ABE8e	54.4%	Richter et al., 2020
		HUDEP2-Δ^G^γ	Lentiviral transduction	−117 *HBG1/2* (HPFH)	hyeA3A-BE4max	50%	Zhang et al., 2020a
	Primary cells	β^−28(*A*>*G*)/^β^−28(*A*>*G*)^ patients fibroblasts and cloned embryos	Plasmid electroporation (fibroblasts) and intracytoplasmic injection of BE3 mRNA (embryos)	*HBB* −28 (A>G) mutation	BE3 YEE-BE3	23%	Liang et al., 2017
		HD HSPCs	mRNA electroporation	−198 and −199 *HBG1/2* (HPFH)	ABE8 variants	50%	Gaudelli et al., 2020
		HD and β-thalassemia patient HSPCs	RNP electroporatin	−114 and −115 *HBG1/2* (HPFH)	hA3A-BE3	20%	Wang et al., 2020
		β^−/^β^−28(*A*>*G*)^ erythroid precursors		*HBB* −28 (A>G) mutation	eA3A(N57G)-BE3 eA3A(N57Q)-BE3	22%	Gehrke et al., 2018
		β^−/^β^−28(*A*>*G*)^ HSPCs		*HBB* −28 (A>G) mutation	eA3A(N57Q)-BE3	68%	Zeng et al., 2020
		SCD and β-thalassemia patient HSPCs^*^		Erythroid-specific *BCL11A* enhancer	eA3A(N57Q)-BE3	86–93%	Zeng et al., 2020
		SCD patient HSPCs^*^		−175 *HBG1/2* (HPFH)	ABE7.10	58%	Mayuranathan et al., 2020
		SCD patient HSPCs^*^	mRNA/RNP electroporation	SCD mutation	ABE8e-NRCH	80/44%	Yen et al., 2020
CAR T-cell therapy	Primary cells	Human primary T-cells	mRNA/RNP electroporation	*TRAC, B2M*, and *PDCD1*	BE4 coBE4	35/80% 90%/ND	Webber et al., 2019
		Human primary T-cells	mRNA electroporation	*B2M, CD7, PDCD1, CIITA, TRAC*, and *CBLB*	ABE8.20-m	98%	Gaudelli et al., 2020

* *tested in vitro and in xenotransplantation experiments in immunodeficient mice*.

*HD, healthy donor; ND, non-determined*.

### Correcting β-Hemoglobinopathy-Causing Mutations With Base Editing

#### Correcting a β-Thalassemia-Causing HBB −28 Mutation Using CBEs

The *HBB* −28 (A>G) mutation is highly prevalent in β-thalassemia patients from China and East Asia. This mutation maps to the ATAA box of the *HBB* promoter and prevents β-globin expression. An HDR-based CRISPR/Cas9 approach was developed to revert this mutation in iPSCs, and restored *HBB* expression in their erythroid progeny (Xie et al., 2014). However, this strategy was not tested in clinically-relevant HSPCs.

Liang et al. used BE3 to correct this mutation in patients' fibroblasts (Liang et al., 2017). However, bystander editing was observed at the −25 position leading to the generation of a mutation causing β-thalassemia in humans. These results highlighted the need to use BEs with a narrower activity window to improve edit precision. Mutation correction was observed also in 23% of human embryos generated by nuclear transfer using a BE with a narrower editing window (YEE-BE3). No bystander edits were observed, suggesting that this BE allows a more precise editing of the *HBB* promoter.

Gehrke et al. compared the efficacy and the precision of BE3, different YE-BE3 variants and eA3A-BE3 in HEK293T cells harboring the *HBB*−28 (A>G) mutation (Gehrke et al., 2018). eA3A-BE3 (containing the N57G amino acid substitution) showed the highest efficacy, followed by BE3 and YE-BE3s. eA3A-BE3 also appeared to be more precise than BE3 and YE-BE3s because of the N57G mutation in A3A that minimizes bystander editing activity. The efficacy and precision of different BEs were also compared in erythroid precursors from a compound heterozygous β-thalassemia patient harboring a deletion in exon 1 in one *HBB* allele and the *HBB* −28 (A>G) mutation in the other allele. eA3A-BE3 and eA3A(N57Q)-BE3 (another BE3 variant with a N57Q mutation in hA3A) preferentially edited the−28 position compared to the −25 position (around 20% of alleles carried only the −28 mutation for both enzymes) and eA3A-BE3 showing the lowest bystander activity. However, eA3A(N57Q)-BE3 was more efficient than eA3A-BE3 at the on-target position. In differentiated erythroid precursors, correction of this mutation by eA3A-BE3 and eA3A(N57Q)-BE3 increased *HBB* expression by 2.6- and 4.0-fold, respectively. Finally, eA3A-BE3 caused off-target edits at one out of six analyzed sites, while eA3A(N57Q)-BE3 caused off-target edits at four of the six sites and with higher frequency than eA3A-BE3. Altogether, these results show that CBEs can be used to correct the *HBB* −28 (A>G) mutation and increase β-globin production in β-thalassemia erythroid cells. However, further studies should be conducted to minimize the off-target effects, while maintaining a high base editing efficiency (see paragraph “BE off-target activity”).

The *HBB* −28 (A>G) mutation was also successfully corrected in HSPCs from a heterozygous β-thalassemia patient with a null β^0^ *HBB* allele and the *HBB* −28 (A>G) mutation in the other allele (Zeng et al., 2020). Electroporation of RNPs containing eA3A(N57Q)-BE3 complexed with the same sgRNA used in the previous study (Gehrke et al., 2018) led to 68% of corrective C>T edits, 28% of non-corrective C>G/A edits, 3.6% of unedited alleles and 14% of bystander edits at position *HBB* −25. This low bystander editing frequency could unlikely lead to the generation of β-thalassemic phenotype. Analysis of single erythroid progenitors demonstrated that corrective C>T edits in position −28 restored β-globin expression. These results demonstrate that base editing can produce efficient and therapeutic edits in primary human HSPCs and, therefore, is a conceivable therapeutic approach to treat β-hemoglobinopathies.

#### Correcting the SCD-Causing β^S^-Globin Allele With ABEs

Miller et al. used novel BE variants to edit the previously inaccessible pathogenic SCD mutation in the *HBB* gene in HEK293T cells (Miller et al., 2020). The mutated allele harbors at position 6 a GTG codon that codes for a valine instead of the wild-type GAG codon translated to a glutamic acid. With the current base editing technology, this A>T mutation cannot be reverted. However, the GTG codon can be converted to a GCG triplet coding for an alanine. This mutation is present in the Makassar allele (HbG) and is non-pathogenic in both heterozygous and homozygous individuals (Viprakasit et al., 2002; Mohamad et al., 2018). Miller et al. tested the ABE-NRRH, ABE-NRTH, and ABE-NRCH variants (compatible with NRRH, NRTH, and NRCH PAMs, respectively), and the previously reported NG-ABEmax [compatible with an NG PAM (Huang et al., 2019)] and sgRNAs targeting protospacer sequences followed by CATG and CACC PAMs in HEK293T cells homozygous for the β^S^-allele. These novel ABEs showed higher on-target base editing activity when using sgRNAs targeting protospacer sequences followed by CACC PAM with ABE-NRCH variant being the most efficient (conversion rate: 41 ± 4%).

A combination of the engineered deaminase of ABE8e and the Cas9n-NRCH led to the creation of ABE8e-NRCH enzyme. This BE efficiently generated 80 and 45% of HbG alleles after RNA or RNP electroporation of SCD HSPCs, respectively. After erythroid differentiation, the high HbG expression (76 and 52% of the total Hb types in samples treated with RNA or RNP electroporation, respectively), and the concomitant decrease of HbS expression, rescued the RBC sickling phenotype. Importantly, editing of the SCD mutation was maintained in xenotransplanted mice (Yen et al., 2020). Altogether, these results show that base editing can be used to modify the SCD-causing β^S^-allele in order to generate a non-pathogenic variant.

### Base Editing Strategies for Reactivating Fetal Hemoglobin to Treat β-Hemoglobinopathies

Correcting the SCD point mutation is a feasible therapeutic approach as all the SCD patients have the same mutation. However, since β-thalassemia is associated with >300 mutations, this approach seems inconceivable to treat this disease as many mutation-specific therapeutic products should be developed. Interestingly, the clinical course of β-hemoglobinopathy patients is ameliorated in the presence of genetic mutations causing a condition termed hereditary persistence of fetal hemoglobin [HPFH (Forget, 1998)]. Therefore, an approach aimed at reactivating the γ-globin genes (*HBG1* and *2*) and fetal hemoglobin (HbF) could represent a universal strategy for treating not only β-thalassemia but also SCD patients. HPFH mutations in the promoter of the γ-globin genes either generate *de novo* DNA motifs recognized by transcriptional activators (e.g., KLF1, TAL1, and GATA1) or disrupt binding sites for transcriptional repressors (e.g., BCL11A and LRF).

Base editing strategies have been developed to reactivate HbF either by generating HPFH mutations or by downregulating the HbF repressor BCL11A via disruption of its erythroid-specific enhancer. It is noticeable that, differently from CRISPR/Cas9 nuclease, base editing allows also the generation of HPFH mutations that create binding sites for transcriptional activators.

#### Inserting HPFH Mutations in the HBG1/2 Promoters

Gaudelli and colleagues designed a sgRNA that allows ABE7.10 to generate a C-to-T conversion at position −198 in both *HBG1* and *HBG2* promoters in HEK293T cells with 29 and 30% of efficiency, respectively (Gaudelli et al., 2017). This point mutation is known to cause HPFH in adults by recruiting the KLF1 transcriptional activator. Similarly, Koblan et al., used ABEmax to install the following HPFH and HPFH-like mutations in HEK293T cells: (1) −175 T>C (generating a binding site for TAL1); (2) −113 A>G (generating a binding site for GATA1); and (3) −116 A>G (HPFH-like mutation in the BCL11A binding site) with efficiencies ranging from 27 to 52% in HEK293T cells (Koblan et al., 2018).

The highly efficient ABE8e variant was also capable of installing HPFH mutations in the *HBG1/2* promoters in HEK293T cells (Richter et al., 2020). Interestingly, both ABE8e and ABEmax could successfully generate the −198 and −175 HPFH mutations, but only ABE8e was capable of simultaneously generating both conversions with a frequency of up to 24%. These results indicate that ABE8e can be used for multiplex base editing. Indeed, the generation of multiple HPFH mutations or the simultaneous targeting of genomic regions involved in *HBG1/2* silencing (e.g., the *HBG1/2* promoters and the *BCL11A* gene) could further increase HbF levels.

The −117 G>A HPFH mutation (disrupting the BCL11A binding site) was inserted in an adult erythroid progenitor cell line (HUDEP2-Δ^G^γ) via lentiviral delivery of hyeA3A-BE4max (Zhang et al., 2020a). This enzyme was generated by inserting the N57G mutation into hyA3A-BE4max, to narrow the editing window and avoid bystander editing that was detrimental on the activity of the *HBG1/2* promoters (Zhang et al., 2020a). An editing frequency of up to 50% led to substantial elevation of γ-globin mRNA expression.

More importantly, HPFH mutations have been inserted using BEs in HSPCs. Wang et al. introduced the −115 C>T and −114 C>T HPFH/HPFH-like mutations (disrupting the BCL11A binding site) in healthy donor and β-thalassemia patient HSPCs via electroporation of RNP containing hA3A-BE3 and a sgRNA targeting the *HBG1/2* promoters (Wang et al., 2020). Editing frequency was ~20% with C>non-T editing events (themselves being HPFH mutations) representing one-fifth of the total edits. HbF reactivation was observed in the erythroid progeny of edited HSPCs. Interestingly, this base editing strategy avoided the deletion of the 5.2-kb region between *HBG1* and *HBG2* promoters. This genomic deletion is commonly observed upon Cas9 nuclease-mediated cleavage of the two identical *HBG1* and *HBG2* promoters and results in the loss of *HBG2* gene expression (Traxler et al., 2016; Li et al., 2018a).

Gaudelli et al. used the novel ABE8s to insert the−198 HPFH mutation (generating a KLF1 binding site) in the *HBG1/2* promoters (Gaudelli et al., 2020). HSPCs derived from healthy donors were electroporated with mRNA encoding either ABE8 or ABEmax and a sgRNA targeting the −198 nucleotide of the *HBG1/2* promoters. ABE8 treatment led to higher editing efficiencies (~50%) compared to ABEmax (~30%) at position −198. Furthermore, only ABE8s were able to simultaneously edit positions −198 and −199. The Authors observed a 3.5-fold average increase in γ-globin expression in erythrocytes differentiated from HSPCs treated with ABE8 compared to mock-treated cells. A statistically significant increase of median γ-globin levels was also observed in all ABE8-treated cells compared to ABEmax-treated samples. These results suggest that simultaneous editing at position −198 and −199 by ABE8s contributed to γ-globin induction.

Recently, the −175 HPFH mutation has been efficiently introduced in up to 58% of *HBG* promoters upon ABE7.10-RNP electroporation of SCD HSPCs. Reactivation of HbF expression was obtained in 60% of erythroid cells differentiated from edited HSPCs (14% expression in control cells). This resulted in a 2-fold decrease in the fraction of sickled RBCs. After xenotransplantation in immunodeficient mice, despite the reduced editing frequency, HbF was detectable in 32% of erythroblasts (Mayuranathan et al., 2020).

#### Disrupting the Erythroid-Specific BCL11A Enhancer

CRISPR/Cas9 nuclease-mediated disruption of the binding site for the GATA1 transcription activator within the *BCL11A* erythroid-specific enhancer is associated with potent *BCL11A* downregulation and γ-globin upregulation. Therefore, this GATA1 binding site represents a potent target for inducing HbF. Ongoing clinical trials aim at evaluating the safety and efficacy of this approach in patients with transfusion dependent β-thalassemia (NCT03655678) and SCD (NCT03745287). One year after cell infusion, the two first patients showed a high editing efficiency, strong HbF de-repression and 100% of F-cells in the peripheral blood resulting in transfusion-independence and elimination of vaso-occlusive crises in the SCD patient (Frangoul et al., 2020) Long-term follow-up studies are necessary to confirm safety and efficacy of this therapeutic strategy (Frangoul et al., 2020).

An alternative approach relies on BEs to precisely edit the GATA1 BS while substantially limiting DSBs. The evolved ABE8e variant was employed in HEK293T cells to install simultaneously two A>G edits in the GATA1 binding site of the *BCL11A* enhancer. ABE8e substantially outperformed ABEmax (54% efficiency for ABE8e vs. 8% for ABEmax) (Richter et al., 2020).

Zeng et al. used RNP containing eA3A(N57Q)-BE3 to achieve high frequency of cytosine base edits at the same GATA1 binding site (86%-93%). This resulted in therapeutically relevant HbF induction in erythroid cells derived from β-thalassemia and SCD patient HSPCs (Zeng et al., 2020). In particular, the erythroid progeny of edited SCD HSPCs exhibited high level of HbF expression (up to 32%), and β-thalassemic erythroid cells showed potent HbF induction that led to improved erythropoiesis. Importantly, xenotransplantation experiments in immunodeficient mice showed efficient C>T editing in *bona fide* human HSCs, while the frequency of C>non-T edits was significantly reduced compared to *in vitro*-treated HSPCs. Finally, multiplex editing of erythroid cells from a β-thalassemia patient to simultaneously disrupt the *BCL11A* erythroid enhancer and correct the *HBB* −28 A>G promoter mutation, led to further improvement of the β-thalassemic phenotype, compared to individual editing of the two regions (*BCL11A* enhancer or *HBB* −28 only).

In conclusion, base editing approaches represent a promising new modality for treating patients with β-thalassemia and SCD by reactivating fetal globin gene expression.

### Developing Safe Allogeneic CAR-T Cell-Based Therapies Using Base Editing

Chimeric antigen receptor (CAR)-T cell therapy is based on the engineering of T-cells to attack tumor cells. The current CAR-T cell-based therapies are effective against hematological malignancies, but limited by their autologous nature (Qasim, 2019; Kim and Cho, 2020). Nuclease-based strategies aimed at inactivating multiple genes involved in alloreactivity allowed the generation of allogeneic CAR-T cells. However, DSBs resulting from multiplex nuclease-based genome editing can lead to large genomic rearrangements such as translocations (Stadtmauer et al., 2020). BEs can be employed to inactivate genes (e.g., by generating premature stop codons or by disrupting splice sites). Thus, BEs have been successfully used to develop safe allogeneic CAR-T cell-based therapies (Webber et al., 2019; Gaudelli et al., 2020), virtually eliminating the genotoxic risks associated to DSBs (Figure 2, Table 1).

Webber et al. exploited CBEs to simultaneously target three loci involved in alloreactivity: the T-cell receptor α constant (*TRAC*) locus, β-2 microglobulin (*B2M*), and programmed cell death 1 (*PDCD1*). The ultimate goal was to generate CD19-targeted CAR-T cells without inducing DSBs and potential translocations (Webber et al., 2019). Targeting each locus separately by electroporating BE4 mRNA and individual sgRNAs (targeting splice donor or acceptor sites) was efficient. However, multiple base editing frequency was modest even when using a higher mRNA dose. RNP delivery of BE4 and more significantly mRNA delivery of a codon-optimized BE4 (coBE4) led to considerably higher efficiencies with 90% of protein loss for all the targets and a proportion of triple knockout cells of up to 90%. Importantly, no translocation event was detected in base-edited T cells compared to samples treated with SpCas9 nuclease inactivating the three targets via DSB generation. Multiplex base editing did not affect cell differentiation, expansion and functionality and cytokine production (Webber et al., 2019).

Similarly, Gaudelli et al. used ABE8s to disrupt genes involved in alloreactivity (*B2M, CD7, PDCD1, CIITA, TRAC*, and *CBLB*) by targeting their splice sites. ABE8.20-m was the best performing enzyme, achieving base editing efficiencies of 98-99% for each of the 6 genes targeted individually and a median protein loss of 60% in primary T cells. ABE8.20-m mRNA electroporation of T cells resulted in efficient multiplex editing of three genes (*B2M, CIITA*, and *TRAC)* (with frequencies >98% for each gene) and concomitant reduced protein expression (Gaudelli et al., 2020).

These studies demonstrate the crucial role of base editing in the development of DSB-free and safe allogeneic CAR T-cell-based therapies.

## Challenges of Base Editing Approaches

### BE Delivery

**Figure 3:**
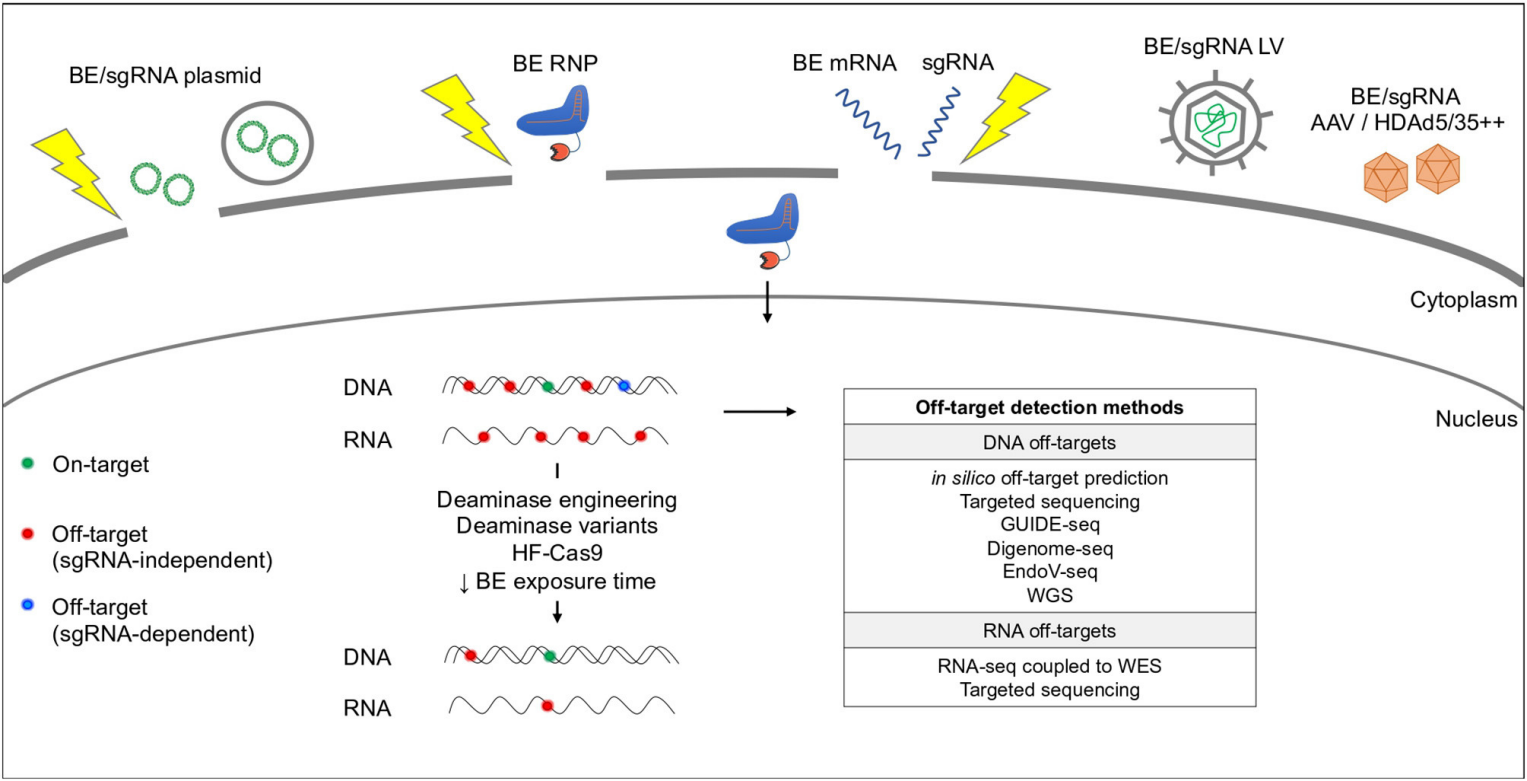
Base editing delivery systems and potential off-target activity. BEs are delivered by plasmid chemical transfection (e.g., lipofectamine) or electroporation (yellow thunder), RNP or mRNA electroporation or LV/AAV transduction. BEs can cause RNA and DNA off-target effects in a sgRNA-independent (red dots) or -dependent (blue dots) manner. Off-target activity can be reduced by modifying the deaminase and/or the Cas9. Current methods used to predict and detect DNA and RNA off-targets are indicated in the table. WGS, Whole Genome Sequencing; WES, Whole Exome Sequencing.

Different methods have been reported to deliver BEs in cell lines and primary cells (Figure 3). Plasmid DNA transfection is an easy, cheap and fast way to produce and deliver BEs to the target cells. Many proof-of-concept studies have used this method to achieve efficient base editing and potentially develop new therapeutic strategies for blood disorders (Gaudelli et al., 2017; Liang et al., 2017; Koblan et al., 2018; Miller et al., 2020; Richter et al., 2020). However, plasmid transfection faces some limitations, such as poor efficiency and toxicity in primary cells (e.g., HSPCs and T cells) (Lattanzi et al., 2019). Moreover, compared to more transient delivery systems (i.e., mRNA and RNP delivery), transfection of BE-expressing plasmids generates more likely off-target effects due to their prolonged expression (Rees et al., 2017).

Lentiviral-mediated BE delivery has also been explored in proof-of-principle studies (Zhang et al., 2020a,b); however, prolonged expression of BEs in hematopoietic cells must be avoided to prevent immune response and off-target editing.

Therefore, transient BE delivery via mRNA or RNP electroporation has been exploited by many research groups to avoid or reduce limitations associated with plasmid and lentiviral delivery. This type of delivery is preferred for the development of clinically-relevant therapeutic strategies (Kouranova et al., 2016).

Several studies aimed at developing treatments for blood disorders have been conducted by delivering mRNAs coding for BEs in T cells and HSPCs [BE3 (Webber et al., 2019), BE4 (Webber et al., 2019), ABEmax (Gaudelli et al., 2020), ABE8 (Gaudelli et al., 2020), and ABE8e-NRCH (Yen et al., 2020)]. Webber et al. showed that codon optimization of BE4 mRNA substantially increases base editing efficiency (Webber et al., 2019). Interestingly, chemical modification of the ABE mRNA (5′capping, uridine depletion and replacement of all remaining uridines with 5-methoxyuridine) and the sgRNA (2′-O-methyl 3′-phosphorothioate modification at first and last three nucleotides) drastically improved ABE protein expression and base editing efficiency in cell lines (Jiang et al., 2020). Therefore, these modifications can potentially increase base editing efficiency in primary cells after BE mRNA delivery.

BE protein production and electroporation in patient-derived HSPCs has been performed successfully for APOBEC3A-based BEs [eA3A-BE3 (Gehrke et al., 2018), eA3A(N57Q)-BE3 (Gehrke et al., 2018; Zeng et al., 2020), and hA3A-BE3 (Wang et al., 2020)]. On the contrary, only Webber et al. have described the delivery of an APOBEC1-based BE as RNP (BE4) in clinically-relevant CD3^+^ T-cells. Furthermore, this study showed that mRNA electroporation outperformed RNP electroporation in terms of base editing efficiency (Webber et al., 2019). However, successful delivery of APOBEC1-based BEs as RNPs was reported in cell lines [BE3 (Kim et al., 2017a; Park et al., 2017; Rees et al., 2017; Yeh et al., 2018), HF-BE3 (Rees et al., 2017), enAsCas12a-BE (Kleinstiver et al., 2019)] although in some cases extensive optimization of the protein production was required (Rees et al., 2017). Regarding ABEs, both ABE7.10 and ABE8e-NRCH were electroporated as RNP complexes in SCD HSPCs, but mRNA electroporation of ABE8e-NRCH led to higher editing efficiencies (Mayuranathan et al., 2020; Yen et al., 2020).

Data on cytotoxicity observed upon BE mRNA or RNP delivery in primary hematopoietic cells are limited (Zeng et al., 2020). Interestingly, two cycles of BE RNP electroporation were required to achieve high frequency of base editing in human HSPCs. This reduced cell viability from 83% (1 round of transfection) to 47% (2 rounds of transfection) and engraftment (Zeng et al., 2020). These results suggest that BE delivery requires further optimization before moving to clinical studies.

As base editing is a recently emerged technology and given the large variety of BEs, BE mRNAs and proteins are not yet commercially available, thus limiting the testing of new therapeutic strategies in primary hematopoietic cells.

Lastly, to overcome the limitations of *ex vivo* HSC-based gene therapy approaches (namely, the loss of the long-term repopulating capacity due to the prolonged culture, and the need for myeloablation and a specialized bone marrow transplantation center), *in vivo* gene therapy strategies have been proposed to deliver BEs to HSCs (Li et al., 2020). To this aim, a suitable *in vivo* delivery system, such as AAV or HDAd5/35++ adenovirus vectors should be used. So far, several studies have established a system to deliver *in vivo* ABEs or CBEs using AAV vectors (Winter et al., 2019; Chen et al., 2020; Hu et al., 2020; Levy et al., 2020). As BEs are large enzymes that cannot be packaged in a single AAV, many groups used a split-intein system based on the use of two AAV vectors harboring the two moieties of a SpCas9-based CBE fused to intein fragments that are reassembled *in vivo* via *trans*-splicing. However, this system is still inefficient, therefore the use of smaller BEs able to be packaged in a single AAV [such as SauriBE4max (Hu et al., 2020)] is preferable. Notably, AAV-mediated delivery specifically to HSCs has not yet been performed and is highly challenging. Intravenous injection of HDAd5/35++ vectors has been used to deliver the CRISPR/Cas9 nuclease system to murine HSCs mobilized in the bloodstream (Li et al., 2020). These vectors can accommodate large expression cassettes, although a selection system needs to be used to reach therapeutically relevant efficiencies of genetic correction in HSCs. Recently, this system has been exploited to deliver into HSCs BEs introducing HPFH point mutations in the *HBG1/2* promoters in a humanized mouse model (Li et al., 2020).

### BE Off-Target Activity

The use of base editing system may lead to undesired DNA and RNA off-target effects. Many efforts have been done to increase the specificity of the Cas9 and the deaminase and eliminate off-targets (Figure 3).

#### DNA Off-Targets

The DNA off-target effects of BEs can be sgRNA-independent or -dependent (Rees et al., 2019).

The sgRNA-independent off-target effects occur at unpredicted sites and are due to the intrinsic DNA affinity of the deaminase domain. Different studies compared the sgRNA-independent off-target activity of CBEs and ABEs and showed a higher frequency of off-targets for CBEs than for ABEs (Zuo et al., 2019; Doman et al., 2020; Lee et al., 2020; Yu et al., 2020). Modifications in the deaminase domain or the use of alternative deaminases allowed the development of CBE variants that exhibit low DNA sgRNA-independent off-target activity and maintain high on-target efficiency (YE1-BE4, R33A-BE4, YE1-BE4-CP1028, and YE1-BE4-NG; (Doman et al., 2020); AmAPOBEC1, SsAPOBEC3B^R54Q^, BE3^R132E^, YE1-BE3, and FE1-BE3; (Yu et al., 2020; Zuo et al., 2020). An alternative way to achieve a high on-target/off-target ratio is to provide the BEs as RNPs. This limits the BE exposure time and reduces the extent of sgRNA-independent off-target editing (Doman et al., 2020). Similarly, BE mRNA delivery decreases off-target editing by limiting BE expression in time (Yu et al., 2020). Therefore, transient BE RNP and mRNA delivery should be preferred compared to plasmid transfection and lentiviral transduction to avoid off-target effects.

Whole genome sequencing is used to evaluate the sgRNA-independent off-target effects, although the coverage is insufficient to detect rare events.

The sgRNA-dependent off-target effects rely on the ability of the Cas9n domain to bind via the sgRNA to genomic sites similar to the on-target site as well as on the presence of an A or a C in the suitable base editing window and in the suitable context for each BE (Gaudelli et al., 2017). Some initial studies suggested that CBEs and ABEs have a lower DNA sgRNA-dependent off-target activity compared to the Cas9 nuclease (Gaudelli et al., 2017; Kim et al., 2017) and that CBEs are in general more prone than ABEs to generate this type of off-target events (Liu et al., 2018; Doman et al., 2020). The use of high-fidelity versions of the Cas9n [e.g., HF-BE3 (Rees et al., 2017), Sniper-Cas9 BE3 (Lee et al., 2018), HF1-eA3A-BE3 and Hypa-eA3A-BE3 (Gehrke et al., 2018)], the BE delivery as RNP (Richter et al., 2020), or even the reduced RNP exposure (Zeng et al., 2020) can minimize the sgRNA-dependent off-target effects.

The *in silico* Cas-off finder software followed by targeted deep sequencing of the predicted off-targets (Gehrke et al., 2018; Wang et al., 2020; Zeng et al., 2020; Zhang et al., 2020a) and experimental methods such as GUIDE-seq (Gehrke et al., 2018; Webber et al., 2019; Gaudelli et al., 2020; Richter et al., 2020; Zeng et al., 2020), Digenome-seq (Kim et al., 2019; Zhang et al., 2020a), and EndoV-seq (Liang et al., 2019; Richter et al., 2020) have been mainly used to evaluate the potential sgRNA-dependent DNA off-target effects of BEs.

#### RNA Off-Targets

BEs may also cause off-target effects at RNA level in a sgRNA-independent manner. The first studies revealed that both CBEs and ABEs can modify the RNA, resulting in tens of thousands of C>U and A>I edits, respectively (Grünewald et al., 2019a; Rees et al., 2019). The RNA edits were spread throughout the transcriptome. To overcome this issue, specific mutations (R33A/K34A) that are known to reduce the RNA C>U base conversion activity of rAPOBEC1 were inserted in CBEs. The resulting BE (BE3-R33A/K34A) presents RNA off-target activity reduced to baseline levels, while maintaining an on-target DNA activity similar to the original BEs (Grünewald et al., 2019a). Furthermore, new rAPOBEC1-containing CBE variants (BE3^R132E^, YE1-BE3, and FE1-BE3) caused a remarkable reduction in the RNA off-target effects (Zuo et al., 2020).

A variety of deaminases and deaminase variants have been used instead of the rAPOBEC1, such as hA3A, eA3A, human AID (hAID), and PmCDA1, to abolish the RNA C-to-U activity of CBEs. hA3A-BE3 showed substantial RNA editing (Grünewald et al., 2019b); however, the use of hA3A harboring amino acid substitutions in the RNA binding domain (R128A) and in the ssDBD (Y130F) abolished RNA off-target effects (Zhou et al., 2019). eA3A induced a number of RNA edits slightly increased compared to controls, while hAID and PmCDA1 had no RNA editing activity (Grünewald et al., 2019b). The use of other deaminases and their simultaneous engineering led to the generation of novel CBEs (backbone of the BE4 with either RrA3F^F130L^, AmAPOBEC1, SsAPOBEC3B^R54Q^, or PpAPOBEC1^H122A/R33A^) with a high ratio of on-target to off-target activity (Yu et al., 2020).

In the case of ABEs, even though the RNA off-target activity was lower in comparison with CBEs, the insertion of point mutations in the TadA domain (E59A or E59Q) and in the engineered TadA^*^ domain (V106W) led to the development of ABE variants (ABEmaxAW and ABEmaxQW) with greatly reduced RNA off-target activity and normal DNA on-target activity (Rees et al., 2019) (Gaudelli et al., 2020)(Richter et al., 2020). Other mutations, such as the F148A mutation in the TadA domain (ABE7.10^F148A^), have also been proved to eliminate the RNA A>I activity of ABEs (Zhou et al., 2019). The removal of the wild-type TadA domain from the classical ABEmax gave rise to a smaller variant (miniABEmax). Its subsequent mutagenesis (in positively charged residues of the engineered TadA^*^ domain that may interact with the phosphate backbone of a nucleic acid) generated miniABEmax^K20A/R21A^ and miniABEmax^V82G^ showing lower off-target activity (Grünewald et al., 2019b).

Notably, some BEs can also modify their own transcripts, leading to a set of heterogeneous base editing proteins. This issue can be eclipsed by employing BE variants with less RNA off-target activity or by using RNPs as a delivery system (Grünewald et al., 2019b).

RNA-seq is commonly used to analyze RNA off-target effects. This analysis should be coupled to whole exome sequencing to exclude that RNA edits are not caused by the editing of the corresponding DNA regions. Alternatively, RNA off-target analysis can be performed by targeting sequencing of RT-PCR amplicons corresponding to commonly edited cellular mRNAs.

Promisingly, in most of the studies focused on the exploitation of BEs for the treatment of a blood disorder, the few detected DNA off-target effects had no predicted functional importance (Gehrke et al., 2018; Webber et al., 2019; Wang et al., 2020; Zeng et al., 2020; Zhang et al., 2020a). In parallel, RNA off-target effects were either undetectable (Webber et al., 2019), or very few (Zhang et al., 2020a), or possibly avoided by deaminase mutations that reduce the RNA editing (Zeng et al., 2020).

## Novel Editing Systems

### Dual-Function BEs

**Figure 4:**
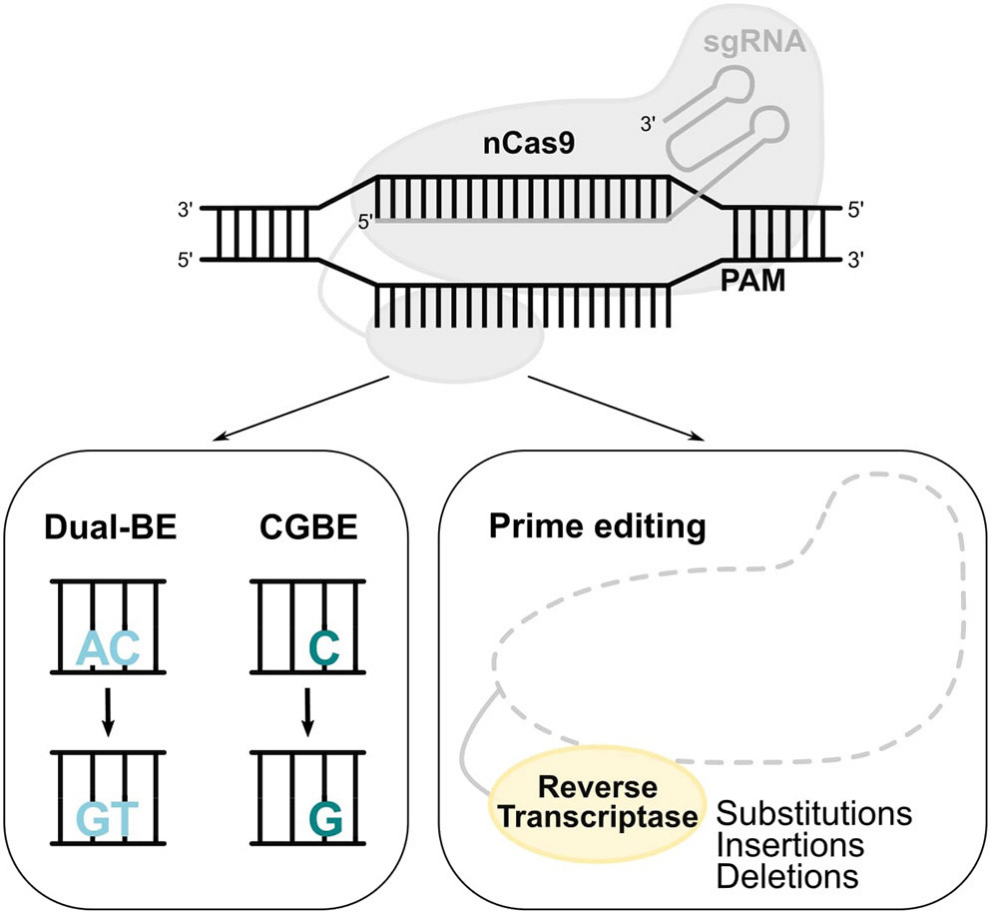
Novel base and prime editors. Novel BEs with a modified deaminase, such as dual functioned BE and CGBE, convert AC-to-GT or CA-to-TG, and C-to-G, respectively. In the prime editing system, a reverse transcriptase uses a pegRNA to install substitutions, insertions, and deletions.

The variety of base editing tools was further expanded in three different studies describing BEs that are able to perform A>G and C>T concurrent substitutions in the same target site (Figure 4). These enzymes [SPACE (Grünewald et al., 2020), Target-ACEmax, ACBEmax (Sakata et al., 2020), and A&C-BEmax (Zhang et al., 2020b)] show either increased or similar editing efficiency compared to the combination of separate ABE and CBE, while displaying similar or even reduced RNA-editing and sgRNA-dependent DNA off-target activity (Grünewald et al., 2020; Sakata et al., 2020; Zhang et al., 2020b). These dual-deaminase BEs expand the targeting spectrum of base editing, allowing more codon changes and TG>CA and CA>TG multi-nucleotide variant modifications, all in the context of a unique protospacer (Grünewald et al., 2020; Sakata et al., 2020; Zhang et al., 2020b). Interestingly, the A&C-BEmax allowed the installation of two different HPFH point mutations in the *HBG1/2* promoters in an adult erythroid progenitor cell lin. These mutations disrupt the BCL11A binding site and generate a DNA motif recognized by the GATA1 transcriptional activator (Zhang et al., 2020b).

### C>G Base Editing

Out of the total pathogenic point mutations [ClinVar database (Rees and Liu, 2018)], 47% can be reverted by ABEs (A>G) and 14% by CBEs (C>T). The generation of a novel BE that performs C>G transversions (CGBE; Figure 4) increased the scope of base editing, allowing the correction of an additional 11% of the total pathogenic point mutations (Rees and Liu, 2018). The development of CGBE was based on: (1) the removal of the UGI from the BE4max architecture to enable the cytosine glycosylation and (2) the addition of an *E.coli*-derived uracil DNA glycosylase that allows C>G transversions (Kurt et al., 2020). CGBE efficiently induced C>G edits with good efficiencies and very few C>T and C>A byproducts. Furthermore, the insertion of the R33A amino acid substitution decreased the RNA off-target edits and the sgRNA-dependent DNA off-target effects. Finally, the targeting range of CGBE was further enlarged by using Cas9 variants with altered or relaxed PAM recognition specificities (Kurt et al., 2020). Overall, CGBE enables 14 different amino acid substitutions that cannot be generated by CBEs or ABEs, and allows the correction of additional disease-causing mutations in both coding and non-coding regions (Kurt et al., 2020).

### Prime Editing

The diversity of base editing tools allows A>G, C>T, and C>G substitutions, with either regular, or more flexible to minimum PAM requirements. The prime editing (PE) system contains a prime editing extended guide RNA (pegRNA)-guided reverse transcriptase instead of a deaminase. The development of PE was a breakthrough as it requires no PAM sequence adjacent to the target site and it can accomplish not only all 12 types of point mutations, but also insertions (of up to 44 bp) and deletions (of up to 80 bp), or even combination of substitutions, insertions and deletions (Figure 4; Anzalone et al., 2019). Importantly, PE showed less DNA off-target activity compared to the CRISPR/Cas9 nuclease system. However, the modestly higher InDel frequency of prime editing compared to base editing should always be taken into consideration and further safety studies need to be performed. A proof-of-principle for the treatment of SCD by PE was provided in HEK293T cells by correcting the disease-causing A>T transversion mutation, which cannot be reverted by the current BEs (Anzalone et al., 2019).

## Conclusions and Perspectives

In conclusion, BEs exhibit plenty of advantages compared to classical approaches of genome editing based on designer nucleases. The low frequency of DSBs generated by BEs is undoubtedly one of the most significant advantages, placing base editing in the top spot amongst the different genome editing tools in terms of safety. Avoiding p53-mediated apoptosis that can result from DSBs formation allows the safe genetic manipulation of p53-sensitive cells, such as HSCs (Milyavsky et al., 2010), and therefore the safe treatment of genetic blood disorders. Moreover, the low frequency of DSB formation prevents the generation of large chromosomal rearrangements, thus maintaining DNA integrity. Importantly, the multiplex editing of two or more loci is feasible with base editing and has been proved very promising in the case of blood disorders. Multiplex base editing led to greater therapeutic effects in β-thalassemic HSPCs edited to simultaneously correct a β-thalassemia-causing mutation and inactivate the *BCL11A* erythroid-specific enhancer. Concomitant editing of 3 loci involved in alloreactivity using BEs allowed the safe production of allogeneic CAR-T cells. In addition, while the CRISPR/Cas9 nuclease system is efficiently used for disrupting genomic regions by generating small InDels (e.g., to disrupt transcription factor binding sites), the base editing system can also be exploited to introduce precise point mutations that either revert disease-causing point mutations or generate *de novo* transcription factor binding sites. These types of modifications can be inserted into the genome through HDR-based strategies, though less efficiently and mainly in target cells that are dividing. Base editing overcomes this obstacle as it is efficacious even in quiescent cells, such as HSCs. Nevertheless, some barriers still exist for base editing, such as the DNA and RNA of-target activity. However, the DNA off-target activity of BEs can be eliminated by using high fidelity Cas enzymes. Similarly, the RNA off-target effects can be abolished by using engineered deaminase variants. Furthermore, the current pool of BEs enables A>G, C>T, and C>G conversions, thus more enzymes need to be created to generate all the different types of conversions, with PE being the current alternative solution to this issue. Last but not least, the delivery of BEs as mRNA or RNP in clinically-relevant cells needs to be further optimized to allow base editing therapeutic approaches to enter the clinical realm.

## Author Contributions

PA, AM, and MB wrote and edited the manuscript. All authors contributed to the article and approved the submitted version.

## Conflict of Interest

The authors declare that the research was conducted in the absence of any commercial or financial relationships that could be construed as a potential conflict of interest.
